# Inferior and Middle Longitudinal Fasciculus and Fornix Support Allocentric Representation

**DOI:** 10.1002/hipo.70031

**Published:** 2025-08-07

**Authors:** Hallvard Røe Evensmoen, Lars M. Rimol, Asta Håberg

**Affiliations:** ^1^ Department of Neuromedicine and Movement Science Norwegian University of Science and Technology (NTNU) Trondheim Norway; ^2^ MiDT National Research Center, Center for Innovation, Medical Equipment and Technology, St. Olav's Hospital Trondheim University Hospital Trondheim Norway; ^3^ Department of Psychology Norwegian University of Science and Technology (NTNU) Trondheim Norway

**Keywords:** cognitive map, hippocampus, memory, spatial learning, visual cortex

## Abstract

If a map‐like representation in the brain originates within a distributed network of brain regions, then this network should be underpinned by white‐matter pathways. To test this, we had 83 young adults learn 15 virtual environments through free exploration, followed by tests of their allocentric and non‐allocentric representation of these environments. Diffusion magnetic resonance imaging (dMRI) ‐ based tractography was used to extract the main white matter tracts in the brain, including the inferior and middle longitudinal fasciculus, the main connections between the temporal lobe and the visual cortices, as well as the fornix, the main output pathway from the hippocampus to the cortex. Higher microstructural integrity in the inferior and middle longitudinal fasciculus and fornix, as indexed by higher FA, was associated with a more accurate scale, rotation, and translation‐invariant allocentric representation of the objects' positional pattern. These findings show that properties of white matter fiber pathways that link visual cortices to the temporal lobe and the temporal lobe to other parts of the brain underpin a person's ability to form successful map‐like representations.

## 1 Introduction

Allocentric representation of space, that is mental representation of the external environment without reference to self‐position, is critical for our way‐finding ability (O'Keefe and Nadel 1978; Tolman 1948). An allocentric representation contains information on relative spatial locations, that is, it contains the positions of landmarks or objects in relation to each other (Ekstrom et al. 2014; Epstein et al. 2017). These *positional patterns* form two‐dimensional overviews of the surrounding environment and constitute fundamental components of cognitive maps. The ability to form allocentric representations seems to depend on an extensive network of brain regions including visual, retrosplenial, parietal, temporal, and prefrontal cortices (Ekstrom et al. 2014, 2017; Epstein et al. 2017; Evensmoen et al. 2021; Huffman and Ekstrom 2019; Weisberg and Ekstrom 2021). Higher global efficacy in a part of this gray matter network that involves the ventral visual stream and the medial temporal lobe has been associated with more accurate allocentric representations (Evensmoen et al. 2021). This suggests allocentric representation in humans depends on white matter pathways connecting the occipital lobe and the temporal lobe, such as the inferior longitudinal fasciculus (ILF) (Catani et al. 2003; Herbet et al. 2018; Latini 2015; Maller et al. 2019) and middle longitudinal fasciculus (MdLF) (Conner et al. 2018; Kalyvas et al. 2020; Latini et al. 2021; Makris et al. 2008, 2017).

The fornix is another white matter tract likely to be involved in allocentric representation. The fornix is a major output pathway from the hippocampus in the medial temporal lobe to the cerebral cortex (via the thalamus) (Benear et al. 2020; Bubb et al. 2018; Cenquizca and Swanson 2007; Daitz and Powell 1954; Duvernoy 2005; Sasson et al. 2013; Saunders and Aggleton 2007). In rats, transection of the fornix results in a reduced ability to learn different spatial environments (Aggleton et al. 1992; Cain et al. 2006; de Bruin et al. 2001; Dumont et al. 2015; Eichenbaum et al. 1990; Ennaceur and Aggleton 1997; Ennaceur et al. 1996; Hudon et al. 2003; Markowska et al. 1989; O'Keefe et al. 1975; Olton et al. 1978; Packard et al. 1989; Packard and McGaugh 1992; Walker and Olton 1979; Warburton and Aggleton 1998; Warburton et al. 1998; Whishaw and Tomie 1997), especially when different starting positions are used (Cain et al. 2006; de Bruin et al. 2001; Eichenbaum et al. 1990; Packard and McGaugh 1992; Warburton and Aggleton 1998; Warburton et al. 1998). This suggests that the fornix is especially important for a flexible representation of an environment. In humans, microstructural properties of the fornix have been associated with the ability to learn the location of a hidden sensor in a virtual environment (Hodgetts et al. 2020), but whether or not the participants used an allocentric strategy was not assessed.

Other brain regions that have been predicted to be important for allocentric representation are the retrosplenial, prefrontal, and parietal cortices (Ekstrom et al. 2017). The most prominent white matter tracts connecting these brain regions as well as the temporal lobe are the cingulum bundle (Bubb et al. 2018). The uncinate fasciculus is another white matter tract that could be involved in allocentric representation as it connects the frontal lobe and the anterior temporal lobe (Von Der Heide et al. 2013). The uncinate fasciculus is involved in associative memory across a range of domains (Alm et al. 2016; Maruta et al. 2010; Metzler‐Baddeley et al. 2011; Thomas et al. 2015; Von Der Heide et al. 2013).

Overall, a key question that remains is whether the ability to construct more accurate allocentric representations is specifically linked to the integrity of white matter tracts in the brain. To investigate this question, we used dMRI‐based tractography (TRACULA) to map the white matter microstructure of 42 major white‐matter tracts in participants who freely explored 15 virtual environments, followed by several tests to assess their cognitive maps. Based on the scores from these tests, we extracted each participant's ability to accurately encode allocentric and non‐allocentric aspects of the environments recently explored, including a scale, rotation, and translation‐invariant allocentric representation of the objects' positional pattern. We predicted that increased microstructural integrity in the inferior and middle longitudinal fasciculus and fornix, and possibly also the cingulum bundle and uncinate fasciculus, would be associated with more accurate allocentric representation of the virtual environments.

## 2 Method

### 2.1 Participants

Seventy‐eight men and five women (18–31 years, mean 23.7 years) with no history of neurological disorders, head trauma, previous or current DSM‐IV axis I diagnosis of psychiatric illness, including substance abuse, were recruited. They were all right‐handed, ascertained with the Edinburgh Handedness Inventory, with a mean score of 88.1% ± 13.9%. The study was approved by the Regional Committee for Medical Research Ethics in central Norway.

### 2.2 MRI Acquisition

Anatomical and diffusion MR images were acquired with a 32‐channel Head Matrix Coil on a 3 T Siemens Skyra scanner (Siemens AG, Erlangen, Germany). Foam pads were used to minimize head motion. The anatomical MRI data was acquired using a *T*_1_‐weighted 3D MPRage sequence (TR = 1900 ms, TE = 3.16 ms, FOV = 256 × 256 × 192 mm, matrix 256 × 256 × 192, giving a resolution of 1.0 × 1.0 × 1.0 mm^3^). The diffusion data was obtained using a single‐shot balanced echo‐planar imaging sequence in 30 noncollinear directions with *b* = 1000 s/mm^2^ and the following parameters: 55 transversal slices, TR 6800 ms, TE 84 ms, FOV 240 × 240 mm, acquisition matrix 96 × 96, slice thickness 2.5 mm, no gap, in‐plane resolution 2.5 × 2.5 mm. For each slice, six images without diffusion weighting (*b* = 0) and 30 images with diffusion gradients were acquired. The DTI sequence was repeated twice for increased signal‐to‐noise ratio. To correct for image distortion caused by magnetic susceptibility artifacts, two additional *b*0 images were acquired with opposite phase–encode polarity (Holland et al. 2010).

### 2.3 Virtual Environmental Learning Paradigm

**FIGURE 1:**
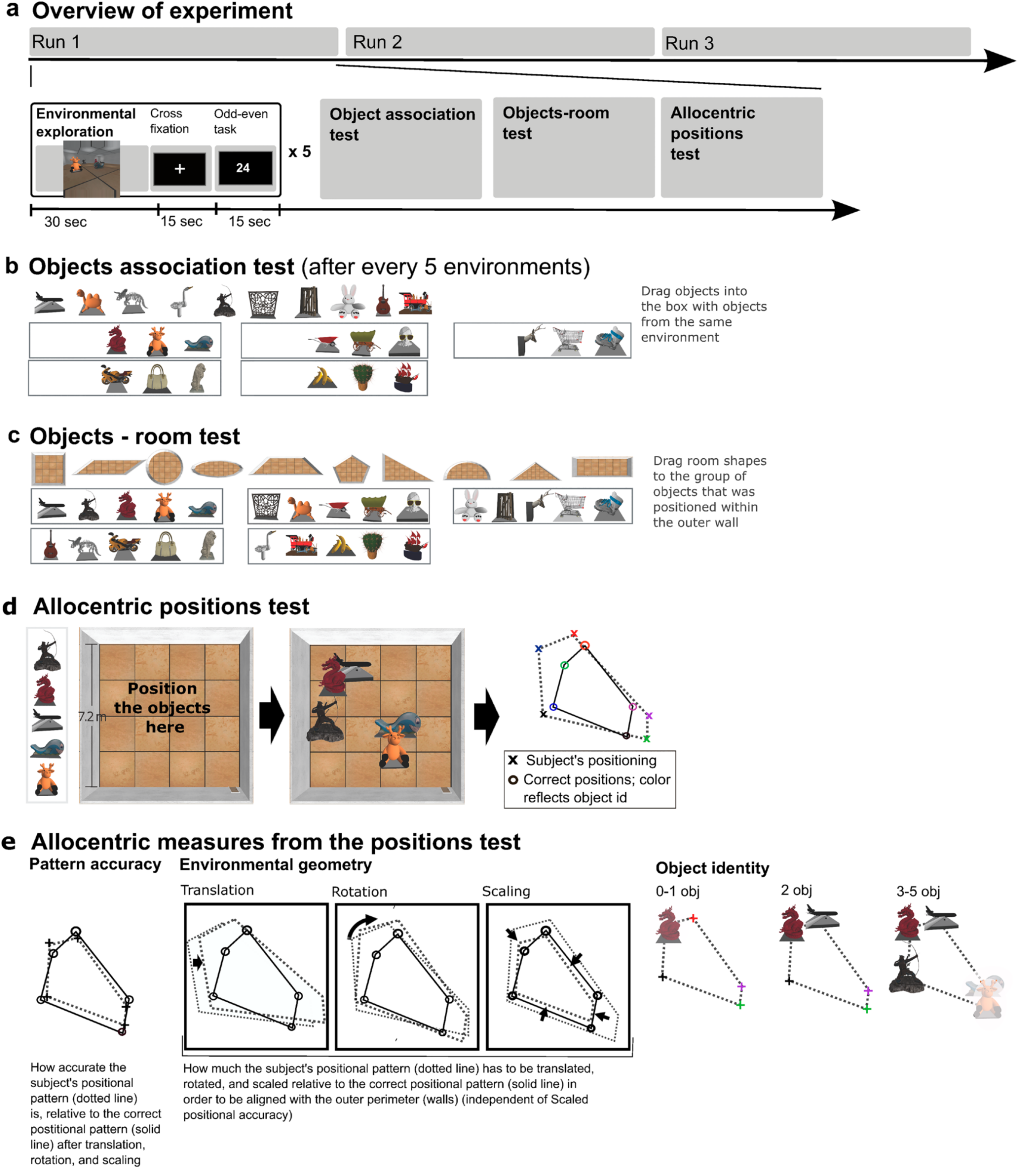
Virtual environmental learning paradigm. (a) Overview of the experiment with the top row showing the number of runs and the bottom row the design within a single run. The participants first freely explored the virtual environment from a first‐person perspective using a joystick to move around (environmental exploration), followed by a post‐exploration encoding period while fixating on a cross and, subsequently, an odd‐even judgment task. After five environments had been presented, the participant's recall was evaluated. (b) The participants first completed the Objects association test, in which the participants grouped together objects from the same environment. (c) Then the participants completed the Objects‐room test, involving assigning a given group of objects to the environment in which they had been presented. (d) Finally, the participants performed the allocentric position test. Left, the participants viewed the environment from a 2D overview and were asked to drag and drop the objects into their correct locations using the joystick. Right: An actual response from one of the participants, with colors indicating object identity. (e) Several allocentric measures were obtained from the Allocentric position test. Left: Pattern accuracy reflects the degree to which the relative positions of the objects (their positional pattern) were correctly recalled when object identity is disregarded and the pattern has been translated, rotated, and scaled relative to the correct positional pattern. Center: Environmental geometry reflects the degree to which the positional pattern, as recalled by the participant, had to be rotated, translated, and scaled to align with the outer wall (independent of pattern accuracy). Right: Object identity reflects how many objects were recalled in their correct positions within the positional pattern (independent of pattern accuracy) (see Method for details).

The participants were first allowed to familiarize themselves with the presentation equipment and the joystick, and then completed practice trials from the different experimental conditions. The participants were then exposed to a total of 15 virtual environments. The paradigm consisted of seven runs, with 5 blocks in each run, and one environment per block. Within each block, there were three periods: environmental exploration (30 s), post‐exploration (with fixation cross; 15 s), and an odd–even task (15 s) (Figure 1a). Environmental exploration took place in a room with a door and an outer perimeter (walls), which had one of 10 geometric shapes. All environments were between 50 and 90 virtual m^2^ large and contained five unique, unrelated objects. Within each environment, the objects were positioned in a unique pattern (Figure 1b). In the environmental exploration period, the participants started at the door of the room (environment) and were free to explore the environment from a first‐person perspective (using a joystick with moving speed set to two virtual m/s) (Figure 1a). After environmental exploration, the participants fixated on a cross on the computer screen while being engaged in non‐stimulus driven encoding (15 s) (Cohen et al. 2015). In the following odd–even task (15 s), the participants were instructed to push the right joystick button when an even number (< 100) appeared on the screen and the left joystick button when an odd number (< 100) appeared (numbers presented at random). The participants were explicitly instructed to focus on getting the odd–even judgments correct. The purpose of the odd–even task was to prevent conscious processing of the episode (Wamsley 2022). The relatively short length of these consolidation periods was based on rest periods lasting only a few seconds benefiting consolidation (Ben‐Yakov et al. 2013; Cohen et al. 2015; Wamsley 2022). The virtual environments were developed in collaboration with Terra Vision AS (Terra Vision, Trondheim, Norway) using the Torque game engine (Garage Games, Eugene, Oregon, US).

Between runs, the participants were given three sets of tests that assessed recall of various spatial and non‐spatial information from each of the five recently learned environments, including an Objects association test, Objects‐room test, and Allocentric positions test (Figure 1b–e). The participants viewed the tests on the computer screen behind the bore opening, and responded by dragging and dropping objects using a joystick while lying in the scanner. The environments were presented in a random sequence across participants, within and between runs. The tests were programmed using HTML and JavaScript (Hansen et al. 2015).

### 2.4 Measures Used to Assess Allocentric and Non‐Allocentric Accuracy

In the Objects‐association test, three of the five objects belonging to an environment were grouped together, and the participant was instructed to select the fourth and fifth object among 10 previously presented objects. The participant's responses were scored as correct or incorrect, according to whether an object was placed in the correct environment. In the following Objects‐room test, the participants were asked to assign a given group of objects to the environment in which they had been presented, based on the geometry of the perimeter of the environment. The participant's responses were scored as correct or incorrect, according to whether a group of objects was placed together with the correct environment. Finally, the participants performed the Allocentric position test, from which several allocentric measures were obtained (detailed below; see Figure 1e). For each environment (block), the five objects belonging to that environment were presented next to a 2D overview of the environment (see Figure 1d), and the participants were asked to drag and drop each object into its original location. Participants were instructed to position each object as accurately as possible. Three allocentric measures were derived: Pattern accuracy, Environmental geometry, and Object identity (see explanation below).

Pattern accuracy indicates whether the participant accurately reproduced the positional pattern of the five objects presented in each block, regardless of the identity of the objects (Figure 1e) (Evensmoen et al. 2015, 2021; Thorp et al. 2024). The participant response (drag‐and‐drop into the 2D overview) was translated so that the geometric center (centroid) of the recalled positional pattern matched the center of the correct positional pattern (as originally presented during the environmental exploration period). Then the recalled positional pattern was rotated into alignment with the correct positional pattern, minimizing the root mean square deviation between the patterns (Kabsch 1976). Next, the recalled positional pattern was scaled (up or down) to minimize the root mean square deviation between the patterns (Umeyama 1991). The purpose of the first two transformations (translation, rotation) was to disentangle the positional pattern as such from its relation to the environment's perimeter (walls of the room). The purpose of scaling was to account for the fact that human spatial representations are sometimes compressed or expanded (Evensmoen et al. 2021; Tversky 1992). After these transformations, Pattern accuracy was obtained as the inverse of the total sum of squares error after all transformations had been performed. That is, for each position in the transformed positional pattern, the squared error was obtained with respect to the closest position in the correct positional pattern, and all such squared errors were summed to obtain the total sum of squares error. This means that Pattern accuracy reflects a scale, rotation, and translation—invariant representation of the objects' positional pattern independent of the identity of the objects. Environmental geometry indicates how accurately the recalled positional pattern was located relative to the perimeter of the environment (Figure 1e). It was defined as the difference between Pattern accuracy and the inverse of the total sum of squares for the recalled positional pattern before translation, rotation, and scaling. Thus, high Environmental geometry implies a low degree of rotation, translation, and scaling relative to the perimeter of the environment. If Pattern accuracy was classified as “failed”, then Environmental geometry was not calculated (see below). Finally, Object identity indicates how accurately the participant recalled which object was placed where within the positional pattern (Figure 1c) (requiring at least a “coarse” level of Pattern accuracy; if Pattern accuracy was “failed”, Object identity was not calculated).

The purpose of including a category of “failed” responses for Pattern accuracy was to be able to identify trials where it was unlikely that allocentric encoding had taken place, such that including these trials would introduce noise into the analyses. “Failed” was defined as “accuracy at or below chance level”. Chance level was estimated by comparing the distribution of scores when a recalled positional pattern was compared to the correct positional pattern, with the distribution of scores when the response from a sub‐group of subjects was compared to the correct positional patterns from an extended set of 120 environments (excluding the correct one) (Evensmoen et al. 2021). The optimal cut‐off value between these two distributions was estimated using R 3.3.3 (R Core Team (2016). R: A language and environment for statistical computing. R Foundation for Statistical Computing, Vienna, Austria. https://www.R‐project.org/) and the pROC package (https://cran.r‐project.org/web/packages/pROC/index.html). We constructed a receiver operating characteristics (ROC) curve by plotting true positive fraction (sensitivity) versus true negative fraction (specificity) for different thresholds (or cut‐off values) between the two distributions (see Figure S1b in (Evensmoen et al. 2021)). The optimal threshold between the two distributions was estimated by minimizing the misclassification fractions, using the best method argument in the pROC package.

### 2.5 MRI Image Preprocessing and Quality Assessment

All MRI raw data was first visually assessed by going through the images slice by slice. Second, image distortions caused by magnetic susceptibility artifacts was estimated in FSL 6.0.3 using the topup command and *b*0 images with opposite phase–encode polarity, exploiting the fact that these two images will have the same distortion of the signal but in opposite directions (Andersson et al. 2003; Graham et al. 2017; Holland et al. 2010; Smith et al. 2004). Third, the eddy command in FSL was used to estimate image distortions induced by eddy currents and motion inside the scanner by registering the diffusion‐weighted *T*_1_ images to the *b =* 0 images (Andersson and Sotiropoulos 2016), and then the diffusion‐weighted *T*_1_ images were corrected for image distortions caused by magnetic susceptibility artifacts (using output from topup) as well as eddy currents and motion inside the scanner in one single resampling.

Next, to be able to estimate the probable relative position of each of the white‐matter pathways with reference to surrounding anatomy (see below), we mapped the anatomical details in the *T*_1_ image to the diffusion‐weighted images using Freesurfer and FSL. First, an automated segmentation and cortical parcellation of *T*_1_‐weighted data were performed for each participant using FreeSurfer 7.4.1 (Dale et al. 1999; Fischl and Dale 2000; Fischl et al. 2002; Fischl, Salat, et al. 2004; Fischl, Sereno, and Dale, 1999; Fischl, Sereno, Tootell, and Dale 1999; Fischl, van der Kouwe, et al. 2004). Second, the diffusion image was registered to the T_1_ image using a boundary‐based cost function with 6° of freedom (rigid) (Greve and Fischl 2009), and then the individual FA map was non‐linearly registered to a FA template using the symmetric normalization from ANTs to make sure that all the brains had the same orientation (Avants et al. 2008; Tustison et al. 2021). These registrations were then used to transform the diffusion‐weighted images and the anatomical segmentations to template space.

### 2.6 Diffusion MRI Tractography

TRACULA (TRActs Constrained by UnderLying Anatomy) in FreeSurfer 7.4.1 was used for automatic reconstruction of white matter tracts from diffusion‐weighted MR images. In TRACULA, global probabilistic tractography is combined with atlas‐derived anatomical prior distributions to propose the probable relative position of the pathway with reference to surrounding anatomy. This means that the software maps the pathways based on where tracts have been manually identified in a control sample at an earlier time point. In doing so, it represents the likelihood that each pathway passes through or lies adjacent to segmentation labels along the predefined pathway's trajectory. TRACULA then applies cortical and subcortical parcellation and segmentation labels from FreeSurfer to reconstruct the different WM pathways (Maffei et al. 2021; Yendiki et al. 2011).

The default tensor fitting and tract reconstruction pipelines using the ball‐and‐stick model were implemented on the pre‐processed data. The ball‐and‐stick model of diffusion is multi‐fibrous and allows for one section of isotropic diffusion and multiple sections of anisotropic diffusion per voxel, which then expresses the diffusion image as a function of the volumes and orientations of these compartments (Behrens et al. 2007). This allows for more than one tract orientation per voxel (Yendiki et al. 2011), and the atlas of anatomical priors obviates the need for manual thresholding or ROI drawing, making TRACULA an ideal tool for tractography analysis of large datasets. To test the hypothesis that proxies of WM integrity from DWI are associated with spatial processing ability, all WM pathways reliably reconstructed in TRACULA were investigated. In addition to the inferior and middle longitudinal fasciculus and fornix, we also included a large set of intrahemispheric tracts primarily as control tracts: arcuate fasciculus, acoustic radiation, anterior thalamic radiation, cingulum bundle, corticospinal tract, extreme capsule, frontal aslant tract, optic radiation, superior longitudinal fasciculus, and uncinate fasciculus. We also included several interhemispheric tracts as control tracts: anterior commissure, corpus callosum body c., corpus callosum body p., corpus callosum body pf., corpus callosum body pm., corpus callosum body t., corpus callosum body genu, corpus callosum rostrum, corpus callosum splenium, and middle cerebellar peduncle. Not included was the cingulum anterior bundle (CAB), due to difficulties obtaining reliable reconstructions of the tract (see (Rimol et al. 2019)). To detect any failed tract reconstruction, we inspected the reconstruction of each tract visually. We found that the reconstruction failed bilaterally for the fornix in four subjects, bilaterally for the corticospinal tract in two subjects, and for the anterior commissure in four subjects.

From each of the tracts, we extracted both the mean fractional anisotropy (FA) and mean diffusivity (MD), “to maximize the specificity and better characterize the tissue microstructure” (Alexander et al. 2007). FA evaluates how anisotropic the diffusion in the tract is, that is how much larger the diffusion is along the tract compared to orthogonal to the tract (Chanraud et al. 2010; Le Bihan et al. 2001; Pierpaoli et al. 1996), and it has been shown that FA increases with increased density of myelinated fibers (Seehaus et al. 2015) and with myelination (Harsan et al. 2006; Song et al. 2002). FA is considered the main DTI proxy for evaluating the integrity of the white matter tracts in the brain (Alexander et al. 2007; Mori and Zhang 2006), and was therefore considered the main DTI measure in this study. FA has a range from 0 (isotropic) to 1 (anisotropic). MD is defined as the mean diffusion along all axes of the tracts (Pierpaoli et al. 1996), and has been shown to decrease with increased density of myelinated fibers (Seehaus et al. 2015).

Finally, partial‐volume fraction effects were estimated for each of the white matter tracts (Vos et al. 2011). First, FreeSurfer was used to estimate the percentage of white matter for each voxel in the brain on an individual level using the t1 image. Second, based on this whole brain white matter map, we used FSL to estimate the average percentage of white matter for the voxels that constituted each of the white matter tracts.

### 2.7 The Relationship Between White Matter Microstructure and Allocentric Representation

To investigate whether there was an association between WM FA and MD and the ability to recall allocentric and non‐allocentric aspects of environments, we employed mixed linear models with maximum likelihood estimates. The data was analyzed in R 4.3.1 (R Core Team (2016). R: A language and environment for statistical computing. R Foundation for Statistical Computing, Vienna, Austria. https://www.R‐project.org/), using the mixed linear model package lme4 (https://cran.r‐project.org/web/packages/lme4/index.html). The R package sjPlot was used for visualization (https://cran.r‐project.org/web/packages/sjPlot/index.html). In these analyses, the mean FA or MD from the WM tracts were the response variable in separate models. The explanatory variables were selected based on whether their inclusion improved the AIC (Akaike information criteria) value of the model by using the buildmer package (https://cran.r‐project.org/web/packages/buildmer/index.html). In addition, we evaluated absolute measures of goodness‐of‐fit to determine whether the included variables were indeed informative (Mac Nally et al. 2018). We also estimated the variation inflation factors for each model in order to evaluate collinearity between the explanatory variables (using the R package car) (https://cran.r‐project.org/web/packages/car/index.html). First, we tested for random intercepts across participants. The fixed effects explanatory variables tested for inclusion in the model were the allocentric and non‐allocentric measures (Pattern accuracy, Environmental geometry, Object identity, Objects association, and Objects‐room association), hemisphere (for all tracts except the interhemispheric tracts), average dropout score (for DWI image slices with excessive intensity drop‐out) (Yendiki et al. 2011), average partial‐volume fraction effect for each white‐matter tract (see above), age, and finally a binary variable indicating whether the participant completed the behavioral part of this study inside the MR scanner or afterwards outside the MR scanner. The significance threshold was corrected for the total number of explanatory variables, across all models, using a 5% False Discovery Rate (FDR).

### 2.8 Data and Software Availability

Due to privacy concerns and official regulations, the ethical and governance approvals for this study do not permit the MRI data to be made available in a public repository. Data in this manuscript can be accessed by qualified investigators after ethical and scientific review (to ensure the data is being requested for valid scientific research) and must comply with the European Union General Data Protection Regulations (GDPR), Norwegian laws and regulations, and NTNU regulations. The completion of a material transfer agreement (MTA) signed by an institutional official will be required. The software developed at NTNU cannot be made freely downloadable according to NTNU's regulations on innovations (patented and not‐patented), but can be made available upon formal agreements between academic institutions.

## 3 Results

### 3.1 Associations Between White Matter Microstructure and Allocentric Accuracy (The Cognitive Map)

**FIGURE 2:**
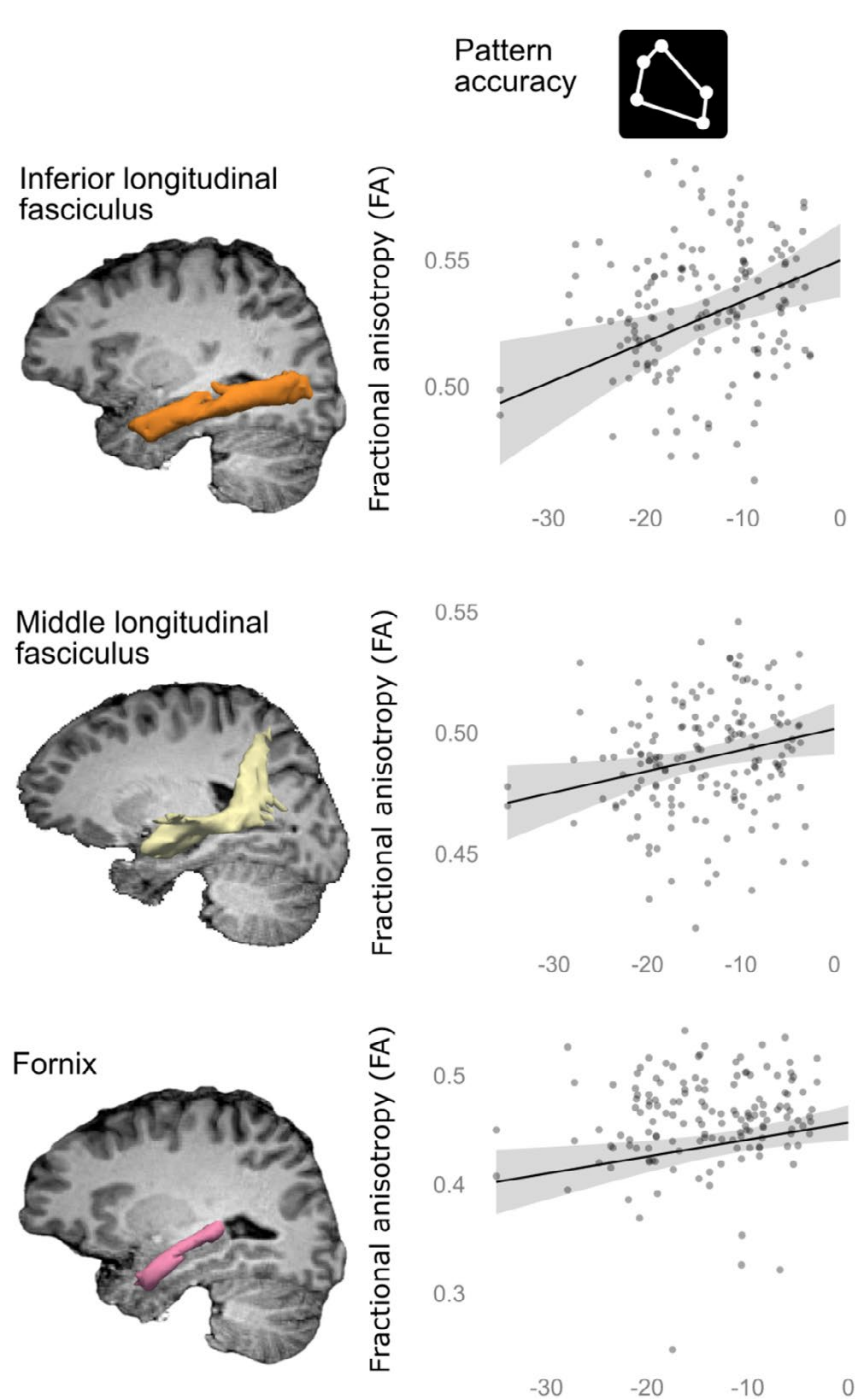
Higher FA was associated with more accurate scale‐invariant allocentric representation. Left, white matter tracts reconstructed by Tracula for one of the subjects. The inferior longitudinal fasciculus (ILF) (upper row, orange), middle longitudinal fasciculus (MdLF) (middle row, yellow), and fornix (lower row, pink) are highlighted. The anatomical *T*_1_ image was used as a background. Right, the plots show the predicted relationship between mean FA and Pattern accuracy from the mixed linear model with 95% confidence intervals (line plots), while controlling for age and DWI image slices with excessive intensity drop‐out. The mixed linear models with mean FA for each of the white matter tracts in the brain as response variable and allocentric and non‐allocentric measures as explanatory variables, revealed that FA in the ILF, MdLF, and fornix were associated with more accurate scale, rotation, and translation‐invariant representation of the objects' allocentric positional pattern (Pattern accuracy). See Tables S1–S4 for more detailed results as well as results for other white matter pathways in the brain. The significance threshold was corrected for the total number of explanatory variables, across all models, using a 5% False Discovery Rate (FDR).

Mixed linear models showed that higher FA in ILF, MdLF, and fornix (indicating higher microstructure integrity) was associated with more accurate scale, rotation, and translation‐invariant allocentric representation of the positional pattern of the objects in the environments (Pattern accuracy) (*t* = 3.1, *p* = 0.016; *t* = 2.5, *p* = 0.049; *t* = 2.7, *p* = 0.042) (Figure 2, Table S1). None of the other allocentric or non‐allocentric measures were associated with FA in ILF, MdLF, or fornix. For most of the other tracts, no relationships between FA and behavioral measures were detected (Tables S1–S4). The exception was lower FA in the frontal aslant tract being associated with more accurate scaling, rotation, and translation of the allocentric positional pattern relative to the outer boundary (Environmental geometry) (*t* = −2.5, *p* = 0.049). No association was observed between MD and any of the allocentric or non‐allocentric measures (Tables S2 and S4). Importantly, the estimated variation inflation factors for each model showed no evidence of a problem with multicollinearity among the explanatory variables in the mixed linear models (Tables S1–S4).

## 4 Discussion

By separating different features recalled from freely explored small virtual environments, we found as hypothesized that the white matter integrity (indexed by FA) in inferior longitudinal fasciculus (ILF), middle longitudinal fasciculus (MdLF), and fornix supported allocentric representations, with a higher FA associated with more accurate scale, rotation, and translation—invariant allocentric representations. FA was not associated with any of the other allocentric aspects of the environments. This suggests that the human brain is optimized for scale, rotation, and translation‐invariant allocentric representations, and that the construction of allocentric representations takes place in a network that involves the white matter pathways between the visual cortices and the temporal lobe, and the temporal lobe and other parts of the brain.

### 4.1 Inferior and Middle Longitudinal Fasciculus

The white matter tracts that connect the visual cortices and the temporal lobe showed a strong association with allocentric accuracy. This underscores the importance of vision and connections between visual regions and the temporal lobe for establishing cognitive maps in humans, and is consistent with the notion that the human ability to use small‐scale maps depends on a high‐resolution visual system (Ekstrom 2015; Muller et al. 2023). In humans, memory for the location of objects has been associated with eye movements (Damiano and Walther 2019; Mou et al. 2006), and vision with no locomotion is sufficient for learning spatial layouts (Muller et al. 2023). Also, non‐human primates rely heavily on vision for allocentric representation, as evidenced by a virtual navigation study revealing that neuronal activity in a part of the ventral visual stream called the parahippocampal cortex (PHC) (Rosenke et al. 2018) conveyed more information about spatial location than activity in other MTL structures (Furuya et al. 2014). Further, hippocampal spatial view cells in monkeys have been shown to encode space within an allocentric framework (Rolls and Wirth 2018), and entorhinal neurons seem to represent space with no locomotion (Killian et al. 2012).

A previous virtual navigation study in humans did not find a relationship between the learning rate of a Morris water maze and FA or MD in the ILF (Hodgetts et al. 2020), but this study did not gauge the participant's 2D map‐like representation of the environment. Our approach involves decomposing the 2D map‐like representation into its constituent parts, allowing for investigation of the neural substrates of allocentric representation independent of confounding factors such as object identity, up‐ or down‐scaling of distances between objects, and relative positioning of objects in relation to the environmental landmarks (such as its perimetry). Thus, we were able to isolate a scale, rotation, and translation—invariant representation of the *positional pattern* of the objects independent of object identity, and show that higher ILF and MdLF integrity (as indexed by FA) was associated with a more accurate representation of such allocentric positional patterns, but not other allocentric or non‐allocentric aspects of the environments.

### 4.2 Fornix

Fornix white matter integrity was associated with a better ability to represent the positional pattern of the objects from an allocentric perspective. Supporting this, fornix transection in rats impaired the ability to use a more flexible and view‐invariant representation (Cain et al. 2006; de Bruin et al. 2001; Eichenbaum et al. 1990; Packard and McGaugh 1992; Warburton and Aggleton 1998; Warburton et al. 1998), but not the ability to use a view‐dependent representation (de Bruin et al. 2001; Eichenbaum et al. 1990) or the ability to recognize objects (Warburton and Aggleton 1998). In primates, the findings are less clear. A previous study in humans found a non‐significant correlation between higher fornix FA and how fast the location of a hidden sensor in a room could be learned (Hodgetts et al. 2020), while others have found that higher fornix FA is associated with increased accuracy of scene and object recollection (Rudebeck et al. 2009) and scene discrimination (Postans et al. 2014). In monkeys, transection of the fornix has been shown to result in a reduced ability to learn the location of an object on a monitor (Buckley et al. 2004, 2008; Bussey et al. 2000; Easton et al. 2002; Gaffan 1994a, 1994b; Kwok and Buckley 2010; Kwok et al. 2015; Parker and Gaffan 1997), and an impaired ability to learn a 3D environment through locomotion (Kwok and Buckley 2006; Murray et al. 1989). Thus, previous studies in primates have not been able to link the fornix to any specific aspects of environmental representation. Our findings indicate that the fornix is especially important for a specific component of the cognitive map, a scale, rotation, and translation‐invariant allocentric representation of the positional pattern of the objects within an environment.

### 4.3 Other Tracts

For the frontal aslant tract, the FA value suggested that lower microstructural integrity was associated with more accurate allocentric representation. One could speculate that subjects with high allocentric proficiency have increased myelination of white matter tracts important for this function, leading to less myelination of the frontal aslant tract. Supporting this, myelination is a high‐energy process (Harris and Attwell 2012; Quintela‐López et al. 2022). Another possibility is that the reduced FA values and increased MD value are related to crossing fibers, as crossing fibers have been shown to reduce FA and increase MD (Poupon et al. 2000; Tuch et al. 2003; Vos et al. 2012). However, the frontal aslant tract does not seem to be the brain tract with the highest number of crossing fibers (Behrens et al. 2007; Schilling et al. 2022), and exclusion ROIs were used in Tracula to remove crossing fibers (Maffei et al. 2021). It is also possible that these white‐matter tracts do support different aspects of the cognitive map, with the frontal aslant tract being part of a network especially important for correctly linking the positional pattern and the environmental geometry. Still, there is little support in the literature for a role of the frontal aslant tract in the construction of cognitive maps (Dick et al. 2019; La Corte et al. 2021). In future studies, it will be important to investigate white matter connectivity in more detail and with more sophisticated methods to increase our understanding of white matter for all aspects of the cognitive map.

We did not find any relationship between reduced MD in any of the other white matter tracts and more accurate allocentric representation. This could be related to the fact that MD only measures the overall diffusion while FA evaluates how much larger the diffusion is along the tract compared to orthogonal to the tract (Chanraud et al. 2010; Le Bihan et al. 2001; Pierpaoli et al. 1996), and a more optimal organization of a white‐matter tract in the brain is expected to lead to both higher diffusion along the tract and lower diffusion perpendicular to the tract. Supporting this, a histological validation study found that high FA values were associated with “high myelin density and a sharply tuned histological orientation profile”, and high mean diffusivity values with “bimodal or diffuse orientation distributions and low myelin density.” (Seehaus et al. 2015).

### 4.4 Limitations

Our study includes very few women as mostly men were recruited. Behaviorally, gender differences have been found for wayfinding (Andersen et al. 2012; Chamizo et al. 2011; Coluccia and Louse 2004; Coutrot et al. 2018; Lawton 2010; Moffat et al. 1998; Wolbers and Hegarty 2010). However, controlling for sex differences in a recent study did not significantly change the relationship between white matter tracts and navigational learning (Hodgetts et al. 2020). Further, for most of the mixed linear models, sex did not explain any variance in white matter microstructure. This suggests that what is observed for men in this paper can be generalized to the population.

## 5 Conclusions

Our findings suggest that the inferior and middle longitudinal fasciculus and fornix are the most important white matter pathways in the human brain for the construction of allocentric representations. This highlights the importance of connectivity between the visual cortices and the temporal lobe and other parts of the brain for the construction of cognitive maps in humans.

## Author Contributions

H.R.E. designed the experiment, conducted the experiments, analyzed the data, and wrote the paper; L.M.R. contributed to the design of the experiment, data collection, analysis of the data, and the writing of the paper; A.H. contributed to the design of the experiment, analysis of the data, and the writing of the paper.

## Conflicts of Interest

The authors declare no conflicts of interest.

## Supporting information


**Data S1:** Supporting Information.

## Data Availability

The data that support the findings of this study are available on request from the corresponding author. The data are not publicly available due to privacy or ethical restrictions.
