# Supplementary material for: Inferior and Middle Longitudinal Fasciculus and Fornix Support Allocentric Representation
Source: Hippocampus. 2025 Aug 7;35(5):e70031. doi: 10.1002/hipo.70031 (PMC12329484; doi:10.1002/hipo.70031)
Supplement: Supplementary file 1 — Data S1: Supporting Information. [file HIPO-35-0-s001.docx]

**Supplementary results**

**Table S1.** FA and allocentric and non-allocentric accuracy for interhemispheric white matter tracts.

|  | **Arcuate fasciculus** | | **Acoustic radiation** | | **Ant. thalamic radiation** | | **Cingulum bundle dorsal** | | **Cingulum bundle ventral** | | **Corticospinal tract** | | **Extreme capsule** | | **Frontal aslant tract** | | **Fornix** | | **Inf. Long. fasciculus** | | **Mid. Long. fasciculus** | | **Optic radiation** | | **Sup. Long. fasciculus 1** | | **Sup. Long. fasciculus 2** | | **Sup. Long. fasciculus 3** | | **Uncinate fasciculus** | |
| --- | --- | --- | --- | --- | --- | --- | --- | --- | --- | --- | --- | --- | --- | --- | --- | --- | --- | --- | --- | --- | --- | --- | --- | --- | --- | --- | --- | --- | --- | --- | --- | --- |
| *Predictors* | *Est.* | *Stat.* | *Est.* | *Stat.* | *Est.* | *Stat.* | *Est.* | *Stat.* | *Est.* | *Stat.* | *Est.* | *Stat.* | *Est.* | *Stat.* | *Est.* | *Stat.* | *Est.* | *Stat.* | *Est.* | *Stat.* | *Est.* | *Stat.* | *Est.* | *Stat.* | *Est.* | *Stat.* | *Est.* | *Stat.* | *Est.* | *Stat.* | *Est.* | *Stat.* |
| (Intercept) | 0.5* | 35 | 0.5* | 195 | 0.5* | 177 | 0.5* | 56 | 0.4* | 11 | 0.6* | 23 | 0.5* | 30 | 0.3 | 1.8 | 0.4* | 17 | 0.6* | 29 | 0.5* | 67 | 0.5* | 209 | 0.6* | 181 | 0.3 | 1.7 | 0.5* | 13 | 0.4* | 21 |
| Hemisphere | 0.0* | 9.5 |  |  |  |  | 0.0* | 9.3 | 0.0* | 2.9 | 0 | 0.9 |  |  |  |  | 0.0* | 6.3 | -0.* | -4.1 | 0.0* | 5.2 |  |  |  |  | -0.0* | -3.2 | 0.0* | 7.5 | 0.0* | 4 |
| Object Association | 0 | -1.6 |  |  |  |  |  |  |  |  |  |  | 0 | -1.9 |  |  |  |  | 0 | -1.5 |  |  |  |  |  |  |  |  |  |  |  |  |
| Pattern Accuracy | 0 | 2.1 |  |  |  |  | 0 | 1.9 |  |  |  |  | 0 | 2 | 0 | 2 | **0.0*** | **2.7** | **0.0*** | **3.1** | **0.0*** | **2.5** |  |  |  |  |  |  | 0 | 2 |  |  |
| Environment Geometry | 0 | -1.7 |  |  |  |  | 0 | -1.6 |  |  |  |  | 0 | -1.9 | **-0.0*** | **-2.5** |  |  |  |  |  |  |  |  |  |  | 0 | -1.6 | 0 | -1.2 |  |  |
| Sex |  |  | 0 | -1.8 |  |  | 0 | -1.7 |  |  |  |  |  |  | 0 | -1.6 |  |  |  |  |  |  |  |  |  |  |  |  |  |  |  |  |
| Objects Room |  |  |  |  |  |  | 0 | -2.1 | -0.1 | -2.4 |  |  |  |  | 0 | -1.8 |  |  |  |  | 0 | -1.8 |  |  | 0 | -1.5 |  |  | 0 | -2.2 |  |  |
| Age |  |  |  |  |  |  |  |  | 0.0* | 2.5 | 0 | -1.5 |  |  | 0 | -1.1 |  |  |  |  |  |  |  |  |  |  |  |  |  |  |  |  |
| Group |  |  |  |  |  |  |  |  |  |  | 0 | 1.3 |  |  | 0 | 1.8 | 0 | 2.3 | 0 | 1.4 |  |  |  |  |  |  |  |  |  |  |  |  |
| Dropout score |  |  |  |  |  |  |  |  |  |  |  |  |  |  | 0.2 | 1.5 |  |  |  |  |  |  |  |  |  |  | 0.2 | 1.5 |  |  |  |  |
| Volume fraction |  |  |  |  |  |  |  |  |  |  |  |  |  |  |  |  | 0 | 1 |  |  |  |  |  |  |  |  | -0.1 | -1.9 |  |  | 0.1 | 2.1 |
| Object Identity Position |  |  |  |  |  |  |  |  |  |  |  |  |  |  |  |  |  |  |  |  |  |  |  |  |  |  |  |  | 0 | -0.9 |  |  |
| **Random Effects** | | | | | | | | | | | | | | | | | | | | | | | | | | | | | | | | |
| σ^2^ | 0 | | 0 | | 0 | | 0 | | 0 | | 0 | | 0 | | 0 | | 0 | | 0 | | 0 | | 0 | | 0 | | 0 | | 0 | | 0 | |
| τ_00_ | 0.00 Id | | 0.00 Id | | 0.00 Id | | 0.00 Id | | 0.00 Id | | 0.00 Id | | 0.00 Id | | 0.00 Id | | 0.00 Id | | 0.00 Id | | 0.00 Id | | 0.00 Id | | 0.00 Id | | 0.00 Id | | 0.00 Id | | 0.00 Id | |
| ICC | 0.74 | | 0.47 | | 0.69 | | 0.67 | | 0.74 | | 0.5 | | 0.67 | | 0.55 | | 0.21 | | 0.75 | | 0.6 | | 0.51 | | 0.66 | | 0.71 | | 0.62 | | 0.66 | |
| N | 83 Id | | 83 Id | | 83 Id | | 83 Id | | 83 Id | | 81 Id | | 83 Id | | 83 Id | | 79 Id | | 83 Id | | 83 Id | | 83 Id | | 83 Id | | 83 Id | | 83 Id | | 83 Id | |
| Observ. | 166 | | 166 | | 166 | | 166 | | 166 | | 155 | | 166 | | 166 | | 147 | | 166 | | 166 | | 166 | | 166 | | 166 | | 166 | | 166 | |
| Marginal R^2^ / Conditional R^2^ | 0.164 / 0.779 | | 0.028 / 0.487 | | 0.000 / 0.688 | | 0.206 / 0.741 | | 0.129 / 0.771 | | 0.033 / 0.518 | | 0.057 / 0.693 | | 0.118 / 0.603 | | 0.249 / 0.406 | | 0.114 / 0.781 | | 0.116 / 0.648 | | 0.000 / 0.505 | | 0.023 / 0.663 | | 0.092 / 0.737 | | 0.159 / 0.684 | | 0.096 / 0.697 | |
|  |  | |  | |  | |  | |  | |  | |  | |  | |  | |  | |  | |  | |  | |  | |  | |  | |
| **VIF(avg(range))** | 1.84(1-2.79) | | NA | | NA | | 1.41(1-1.96) | | 1(1-1) | | 1.03(1-1.04) | | 2.1(1.7-2.8) | | 1.8(1.1-3.4) | | 1.3(1.1-1.5) | | 1.64(1-1.97) | | 1.12(1-1.17) | | NA | | NA | | 1.06(1-1.11) | | 1.44(1-2.13) | | 1.4(1.4-1.4) | |

Fractional anisotropy (FA, range from 0 to 1) of the white matter tracts was used as the dependent variable in (mixed) linear models. Independent variables were selected on the basis of whether their inclusion improved the Akaike information criteria value of the model. First, we tested for random intercepts across participants. The fixed effects independent variables tested for inclusion were the allocentric and non-allocentric measures (Pattern accuracy, Environmental geometry, Object identity, Objects association, and Objects-room association), hemisphere, average dropout score (for DWI image slices with excessive intensity drop-out), average partial-volume fraction effect for each white-matter tract, age, and finally a binary variable indicating whether the participant completed the behavioral part of this study inside the MR scanner or afterwards outside the MR scanner. Statistics is a t-value that reflects the significance of the estimates. * P < 0.05, corrected for multiple comparisons using a 5% False Discovery Rate (FDR).

**Table S2.** MD and allocentric and non-allocentric accuracy for interhemispheric white matter tracts.

|  | **Arcuate fasciculus** | | **Acoustic radiation** | | **Ant. thalamic radiation** | | **Cingulum bundle dorsal** | | **Cingulum bundle ventral** | | **Corticospinal tract** | | **Extreme capsule** | | **Frontal aslant tract** | | **Fornix** | | **Inf. Long. fasciculus** | | **Mid. Long. fasciculus** | | **Optic radiation** | | **Sup. Long. fasciculus 1** | | **Sup. Long. fasciculus 2** | | **Sup. Long. fasciculus 3** | | **Uncinate fasciculus** | |
| --- | --- | --- | --- | --- | --- | --- | --- | --- | --- | --- | --- | --- | --- | --- | --- | --- | --- | --- | --- | --- | --- | --- | --- | --- | --- | --- | --- | --- | --- | --- | --- | --- |
| *Predictors* | *Est.* | *Stat.* | *Est.* | *Stat.* | *Est.* | *Stat.* | *Est.* | *Stat.* | *Est.* | *Stat.* | *Est.* | *Stat.* | *Est.* | *Stat.* | *Est.* | *Stat.* | *Est.* | *Stat.* | *Est.* | *Stat.* | *Est.* | *Stat.* | *Est.* | *Stat.* | *Est.* | *Stat.* | *Est.* | *Stat.* | *Est.* | *Stat.* | *Est.* | *Stat.* |
| (Intercept) | 0* | 230 | 0.* | 27 | 0.0 * | 197 | 0.0 * | 240 | 0.0 * | 128 | 0.0* | 13 | 0.0* | 88 | 0.0* | 15 | 0 | -0.1 | 0.0* | 28.9 | 0.0 * | 30 | 0.0* | 16 | 0.0* | 253 | 0.0 * | 235 | 0.0* | 212 | 0.0* | 25 |
| Objects Room |  |  | 0 | 1.3 |  |  |  |  | 0 | 1.6 | 0 | 2 |  |  |  |  |  |  | 0 | 1.5 | 0 | 1.3 | 0 | 1.2 |  |  |  |  |  |  |  |  |
| Sex |  |  | 0 | 2 |  |  |  |  |  |  |  |  |  |  |  |  |  |  |  |  |  |  |  |  |  |  |  |  |  |  | 0 | 1.4 |
| Age |  |  | 0 | -1.7 |  |  |  |  |  |  | 0 | -1.5 |  |  |  |  |  |  | 0 | -2.4 | 0 | -1.9 | 0 | -1.8 |  |  |  |  |  |  | 0 | 0.7 |
| Hemisphere |  |  |  |  | -0.0* | -3.6 | 0.0 * | 6.8 | 0 | -2.4 | 0 | -1.6 | 0 | -1.7 | 0.0* | 3.2 |  |  | -0.0* | -2.8 |  |  | -0.0 * | -6 | -0.0 * | -6.4 | 0.0 * | 4.1 | -0.0 * | -3.4 | -0.0* | -2.9 |
| Volume fraction |  |  |  |  |  |  |  |  |  |  | 0 | 1.3 |  |  |  |  |  |  |  |  |  |  | 0.0 * | 2.7 |  |  |  |  |  |  | 0 | -2.2 |
| Group |  |  |  |  |  |  |  |  |  |  |  |  | 0 | -1.1 |  |  |  |  |  |  |  |  |  |  |  |  |  |  |  |  | 0 | -1.5 |
| Object Identity Position |  |  |  |  |  |  |  |  |  |  |  |  |  |  | 0 | 1.5 |  |  |  |  |  |  |  |  |  |  |  |  |  |  |  |  |
| Dropout score |  |  |  |  |  |  |  |  |  |  |  |  |  |  |  |  | 0 | 2 |  |  |  |  |  |  |  |  |  |  |  |  |  |  |
| **Random Effects** | | | | | | | | | | | | | | | | | | | | | | | | | | | | | | | | |
| σ^2^ | 0 | | 0 | | 0 | | 0 | | 0 | | 0 | | 0 | | 0 | | 0 | | 0 | | 0 | | 0 | | 0 | | 0 | | 0 | | 0 | |
| τ_00_ | 0.00 Id | | 0.00 Id | | 0.00 Id | | 0.00 Id | | 0.00 Id | | 0.00 Id | | 0.00 Id | | 0.00 Id | | 0.00 Id | | 0.00 Id | | 0.00 Id | | 0.00 Id | | 0.00 Id | | 0.00 Id | | 0.00 Id | | 0.00 Id | |
| ICC | 0.88 | | 0.64 | | 0.88 | | 0.83 | | 0.76 | | 0.59 | | 0.85 | | 0.89 | | 0.59 | | 0.85 | | 0.84 | | 0.74 | | 0.91 | | 0.89 | | 0.74 | | 0.86 | |
| N | 83 Id | | 83 Id | | 83 Id | | 83 Id | | 83 Id | | 81 Id | | 83 Id | | 83 Id | | 79 Id | | 83 Id | | 83 Id | | 83 Id | | 83 Id | | 83 Id | | 83 Id | | 83 Id | |
| Observ. | 166 | | 166 | | 166 | | 166 | | 166 | | 155 | | 166 | | 166 | | 147 | | 166 | | 166 | | 166 | | 166 | | 166 | | 166 | | 166 | |
| Marginal R^2^ / Conditional R^2^ | 0.000 / 0.879 | | 0.071 / 0.666 | | 0.009 / 0.885 | | 0.044 / 0.841 | | 0.036 / 0.768 | | 0.077 / 0.618 | | 0.016 / 0.852 | | 0.032 / 0.893 | | 0.041 / 0.610 | | 0.089 / 0.867 | | 0.058 / 0.849 | | 0.148 / 0.778 | | 0.021 / 0.916 | | 0.011 / 0.894 | | 0.018 / 0.747 | | 0.090 / 0.870 | |
|  |  | |  | |  | |  | |  | |  | |  | |  | |  | |  | |  | |  | |  | |  | |  | |  | |
| **VIF(avg(range))** | NA | | 1.02(1.01-1.03) | | NA | | NA | | 1(1-1) | | 1.01(1.01-1.02) | | 1(1-1) | | 1(1-1) | | NA | | 1(1-1) | | 1(1-1) | | 1.03(1.01-1.06) | | NA | | NA | | NA | | 1.34(1.04-1.71) | |

Mean diffusivity (MD) of the white matter tracts was used as the dependent variable in (mixed) linear models. Independent variables were selected on the basis of whether their inclusion improved the Akaike information criteria value of the model. First, we tested for random intercepts across participants. The fixed effects independent variables tested for inclusion were the allocentric and non-allocentric measures (Pattern accuracy, Environmental geometry, Object identity, Objects association, and Objects-room association), hemisphere, average dropout score (for DWI image slices with excessive intensity drop-out), average partial-volume fraction effect for each white-matter tract, age, and finally a binary variable indicating whether the participant completed the behavioral part of this study inside the MR scanner or afterwards outside the MR scanner. * P < 0.05, corrected for multiple comparisons using a 5% False Discovery Rate (FDR).

**Table S3.** FA and allocentric and non-allocentric accuracy for interhemispheric white matter tracts.

|  | **Ant. commissure** | | **Corpus callosum body c.** | | **Corpus callosum body p.** | | **Corpus callosum body pf.** | | **Corpus callosum body pm.** | | **Corpus callosum body t.** | | **Corpus callosum body genu** | | **Corpus callosum rostrum** | | **Corpus callosum splenium** | | **Middle cerebellar peduncle** | |
| --- | --- | --- | --- | --- | --- | --- | --- | --- | --- | --- | --- | --- | --- | --- | --- | --- | --- | --- | --- | --- |
| *Predictors* | *Estimates* | *Statistic* | *Estimates* | *Statistic* | *Estimates* | *Statistic* | *Estimates* | *Statistic* | *Estimates* | *Statistic* | *Estimates* | *Statistic* | *Estimates* | *Statistic* | *Estimates* | *Statistic* | *Estimates* | *Statistic* | *Estimates* | *Statistic* |
| (Intercept) | 0.3 * | 7.7 | 0.6 * | 121.6 | 0.6 * | 122.4 | 0.6 * | 26.8 | 0.6 * | 85.1 | 0.6 * | 66.7 | 0.6 * | 160.8 | 0.5 * | 10.1 | 0.5 * | 8.6 | 0.5 * | 21 |
| Sex | -0.1* | -4.3 | 0 | -1.6 |  |  | 0 | -1.6 | 0 | -2.1 |  |  |  |  |  |  |  |  |  |  |
| Group | 0.0 * | 3.4 |  |  |  |  |  |  |  |  |  |  |  |  |  |  |  |  |  |  |
| Object Association | 0 | 2.6 |  |  |  |  |  |  |  |  |  |  |  |  |  |  |  |  | 0 | -1.4 |
| Environment Geometry | 0 | -1.5 |  |  |  |  | 0 | -2 | 0 | -1.5 |  |  |  |  |  |  |  |  | 0 | -1.8 |
| Objects Room |  |  | 0 | -1.9 | 0 | -2 | 0 | -2.3 | 0 | -2.6 | 0 | -1.8 |  |  |  |  | 0 | -1.1 |  |  |
| Age |  |  |  |  |  |  | 0 | -1.5 |  |  |  |  |  |  | 0 | -1.5 |  |  |  |  |
| Pattern Accuracy |  |  |  |  |  |  | 0 | 1.4 |  |  | 0 | 1.7 |  |  |  |  |  |  | 0 | 1.6 |
| Volume fraction |  |  |  |  |  |  |  |  |  |  |  |  |  |  | 0.2* | 3.2 | 0.2 | 2.1 |  |  |
| Observations | 79 | | 83 | | 83 | | 83 | | 83 | | 83 | | 83 | | 83 | | 83 | | 83 | |
| R^2^ / R^2^ adjusted | 0.234 / 0.193 | | 0.061 / 0.037 | | 0.046 / 0.035 | | 0.134 / 0.078 | | 0.122 / 0.089 | | 0.054 / 0.031 | | 0.000 / 0.000 | | 0.123 / 0.101 | | 0.074 / 0.051 | | 0.054 / 0.018 | |
|  |  | |  | |  | |  | |  | |  | |  | |  | |  | |  | |
| **VIF (avg(range))** | 1.56(1.29-1.84) | | 1.03(1.03-1.03) | | NA | | 1.42(1.02-1.98) | | 1.09(1.04-1.13) | | 1.17(1.17-1.17) | | NA | | 1.04(1.04-1.04) | | 1.02(1.02-1.02) | | 2.12(1.71-2.79) | |

Fractional anisotropy (FA, range from 0 to 1) of the white matter tracts was used as the dependent variable in (mixed) linear models. Independent variables were selected on the basis of whether their inclusion improved the Akaike information criteria value of the model. The fixed effects independent variables tested for inclusion were the allocentric and non-allocentric measures (Pattern accuracy, Environmental geometry, Object identity, Objects association, and Objects-room association), average dropout score (for DWI image slices with excessive intensity drop-out), average partial-volume fraction effect for each white-matter tract, age, and finally a binary variable indicating whether the participant completed the behavioral part of this study inside the MR scanner or afterwards outside the MR scanner. Statistics is a t-value that reflects the significance of the estimates. * P < 0.05, corrected for multiple comparisons using a 5% False Discovery Rate (FDR).

**Table S4.** MD and allocentric and non-allocentric accuracy for interhemispheric white matter tracts.

|  | **Ant. commissure** | | **Corpus callosum body c.** | | **Corpus callosum body p.** | | **Corpus callosum body pf.** | | **Corpus callosum body pm.** | | **Corpus callosum body t.** | | **Corpus callosum body genu** | | **Corpus callosum rostrum** | | **Corpus callosum splenium** | | **Middle cerebellar peduncle** | |
| --- | --- | --- | --- | --- | --- | --- | --- | --- | --- | --- | --- | --- | --- | --- | --- | --- | --- | --- | --- | --- |
| *Predictors* | *Estimates* | *Statistic* | *Estimates* | *Statistic* | *Estimates* | *Statistic* | *Estimates* | *Statistic* | *Estimates* | *Statistic* | *Estimates* | *Statistic* | *Estimates* | *Statistic* | *Estimates* | *Statistic* | *Estimates* | *Statistic* | *Estimates* | *Statistic* |
| (Intercept) | 0.0* | 25.1 | 0.0* | 108.8 | 0.0* | 28 | 0.0* | 72.3 | 0.0* | 87 | 0.0* | 28.7 | 0.0* | 181.1 | 0.0* | 21.4 | 0.0* | 27.3 | 0.0* | 58.5 |
| Age | 0 | -1.7 |  |  | 0 | -1.7 |  |  |  |  | 0 | -1.9 |  |  |  |  | 0 | -1.8 |  |  |
| Group | 0 | -1.6 |  |  |  |  |  |  | 0 | 1.4 |  |  |  |  |  |  |  |  | 0 | -1.1 |
| Environment Geometry |  |  | 0 | 1.7 |  |  | 0 | 1.6 |  |  |  |  |  |  |  |  |  |  |  |  |
| Pattern Accuracy |  |  | 0 | -2.2 |  |  | 0 | -1.4 |  |  |  |  |  |  |  |  |  |  |  |  |
| Objects Room |  |  |  |  | 0 | 1.2 | 0 | 1.5 |  |  | 0 | 1.4 |  |  |  |  |  |  |  |  |
| Volume fraction |  |  |  |  |  |  |  |  |  |  |  |  |  |  | 0 | -2.9 |  |  |  |  |
| Observations | 79 | | 83 | | 83 | | 83 | | 83 | | 83 | | 83 | | 83 | | 83 | | 83 | |
| R^2^ / R^2^ adjusted | 0.085 / 0.061 | | 0.059 / 0.035 | | 0.056 / 0.033 | | 0.052 / 0.016 | | 0.025 / 0.013 | | 0.069 / 0.046 | | 0.000 / 0.000 | | 0.092 / 0.081 | | 0.039 / 0.027 | | 0.014 / 0.002 | |
|  |  | |  | |  | |  | |  | |  | |  | |  | |  | |  | |
| **VIF (avg(range))** | 1.05(1.05-1.05) | | 1.64(1.64-1.64) | | 1(1-1) | | 1.61(1.21-1.95) | | NA | | 1(1-1) | | NA | | NA | | NA | | NA | |

Mean diffusivity (MD) of the white matter tracts was used as the dependent variable in (mixed) linear models. Independent variables were selected on the basis of whether their inclusion improved the Akaike information criteria value of the model. The fixed effects independent variables tested for inclusion were the allocentric and non-allocentric measures (Pattern accuracy, Environmental geometry, Object identity, Objects association, and Objects-room association), average dropout score (for DWI image slices with excessive intensity drop-out), average partial-volume fraction effect for each white-matter tract, age, and finally a binary variable indicating whether the participant completed the behavioral part of this study inside the MR scanner or afterwards outside the MR scanner. * P < 0.05, corrected for multiple comparisons using a 5% False Discovery Rate (FDR).
